# Automaticity: schema modes in addiction

**DOI:** 10.3389/fpsyt.2023.1158067

**Published:** 2023-10-18

**Authors:** Michiel Boog, Helen Tibboel

**Affiliations:** ^1^Department of Addiction and Personality, Antes Mental Health Care, Rotterdam, Netherlands; ^2^Erasmus School of Social and Behavioural Sciences – Clinical Psychology, Rotterdam, Netherlands

**Keywords:** schema mode, schema therapy, addiction, substance use disorder, automaticity

## Abstract

Automaticity is a hallmark of substance use disorder. In Schema Therapy (an evidence-based form of psychotherapy, that has also been applied to substance use disorders), automaticity appears to be a relevant variable. However, the role of automaticity in Schema Therapy has never been made explicit. In the present article, we investigate the role of automaticity in schema modes and its role in different phases in Schema Therapy for substance use disorders. In performing this investigation, we facilitate a better understanding of the working mechanisms of Schema Therapy, and, vice versa, suggest an alternative understanding of automaticity in substance use disorders. We suggest that the automatic use of substances is way of coping with schemas and, therefore, is the consequence of schema mode activity. In the article, four characteristics of automaticity (unconscious, uncontrollable/uncontrolled, efficient, fast) are translated to schema modes. Subsequently, a Schema Therapy case of a patient suffering from an alcohol use disorder and a narcissistic personality disorder is discussed, focusing on the four facets of automaticity. Last, implications for theory, clinical practice and future research are discussed.

## Introduction

Several prominent psychological models of addiction agree that substance use disorder (SUD) is characterized by automaticity [e.g., (1, 2)]. Whereas these theories are not without their limitations [e.g., (3, 4)], they have inspired many researchers, leading to an extensive literature on this topic [e.g., (1, 2, 5)]. For instance, habit theories of addiction suggest that drug cues automatically elicit a response that involves approaching and consuming the drug [e.g., (5–8)]. On a related note, dual-process views [e.g., (9)] emphasize that addiction is the result of the gradually increasing influence of automatic, rather than controlled, processes. These theories also suggest that drug cues automatically elicit responses. They also suggest that these responses go beyond automatic approach [e.g., (10)] and also involve automatic attentional biases [e.g., (11, 12)] and memory biases [e.g., (13)]. Automaticity thus seems crucial for the development and maintenance of addiction [e.g., (14–16)], but what do we mean when we regard a process as automatic?

In what Moors [(17); see also (18)] describes as the componential view on automaticity, automaticity consists of different features. According to this view, there are different automaticity criteria, and importantly, automaticity is not described as an all-or-none phenomenon. Rather, a process can be regarded as automatic in some ways (e.g., it might meet some of the criteria, but not others), and to some extent (e.g., it might not meet these criteria completely but only to some degree). The following criteria are put forward: unconscious, uncontrolled/uncontrollable, efficient, and fast. Regarding the lack of consciousness, Moors explains that a person can be conscious of different aspects of a process: the input, the output, and the transition from input to output. If consciousness regarding one or more of these components is missing, the process is regarded as unconscious. Note that consciousness can thus be regarded as gradual: there can be a complete lack of consciousness regarding all components of a process, or a person can lack consciousness regarding only some components of a process.

A process is regarded as uncontrolled or uncontrollable when a person cannot choose to engage in or to stop the act (17). Again, different conditions need to be met for a process to be controlled or controllable: a person needs to have an intention to attain a specific goal by engaging in a specific process; they need to attain this goal; and there needs to be a causal relation between the intention and the attainment of the goal. If one of these components is missing, the process is considered to be uncontrolled or uncontrollable. Again, we note that controllability is therefore never an all-or-none phenomenon: it is possible that all components of controllability are absent, or only some of them.

When it comes to efficiency, Moors explains that a process is efficient when the process happens without requiring much attentional or cognitive resources. Efficiency thus means that if the attentional resources of a person are depleted, for instance because they are tired, intoxicated, or are currently executing a demanding task, the process is still executed. It is important to note that efficiency is also a gradual phenomenon. Because there are large interindividual differences in attentional capacity [e.g., (19, 20)] we recommend that, when reflecting on the automaticity of a specific process for a specific person, we consider the relative nature: is the process more or less efficient in one condition compared to another?

Regarding the final component of automaticity, Moors suggests that a process is fast when it requires little time to be activated or when it has a short duration. Just like the previous components of automaticity, it is gradual. And because speed is also characterized by interindividual differences, (19), we suggest it is important to keep the relativity in mind when we examine whether the speed of a process has changed for a specific person.

### Schema therapy and automaticity

In the Schema Therapy (ST) model, automaticity appears to be a relevant variable. However, it seems to be so self-evident, that it is not described thoroughly. ST is an evidence-based form of psychotherapy for individuals suffering from personality disorders (21, 22), although in recent years ST has expanded its reach to several other disorders, like substance use disorders (23–25). The core concepts of ST are schemas and schema modes. Schemas are dysfunctional mental representations that have developed in childhood, and that consist of thoughts, emotions, memories and bodily sensations (26, 27). They develop in interplay between temperamental factors and adverse external childhood factors, like, for instance, abuse and neglect. Schemas distort perception and persist throughout life. The concept of schema modes was developed to come to more parsimonious ST conceptualizations for patients with severe pathology (28). Schema modes are the states of being that result from one or more activated schemas, and a related coping response. Schema modes include behavior (next to cognitions and emotions), in contrast to schemas (26). The triggering of schemas and the activation of modes seem to be highly automatic. Humans are usually not aware of the fact that, for example, their Defectiveness/Shame schema is triggered at a birthday party, or that their Detached Protector mode is activated in a job interview. Interestingly, it seems like automaticity is such a central, obvious process in ST, that it is scarcely reflected upon. If ST authors mention automaticity, it is usually (often casually) in the context of modes (schema coping). For example, Arntz et al. state [(29), pp. 1008–1,009]: “Moreover, according to the original theory coping responses are generally automatic and people are not necessarily making a conscious decision for them (p. 1008–1,009).” And Rafaeli et al. (30, p. 268): “But, whereas child modes (and particularly the Vulnerable Child) capture the helpless and muted emotional reactions of the child, coping modes develop out of a child’s basic survival operations: they are primarily automatic adaptation-promoting measures taken in order to survive in an emotionally negligent or otherwise noxious environment.” To our knowledge, no researchers or clinicians have theorized on the exact role of automaticity in ST. As a result, it remains, for instance, unclear in which way and to what extent schema modes are automatic, and how this affects behavior. This is remarkable, because the whole process of ST seems to be based on uncovering the underlying roots of undesirable behavior, i.e., disrupting and changing automatic behavior. It seems like automaticity is an invisible cornerstone of ST. Whereas the concept of automaticity has often been applied to relatively low-level associative processes (e.g., priming tasks, go no-go tasks, implicit association tasks), this concept has recently been applied to more complex, goal-driven, and propositional processes as well [e.g., (31, 32)]. Our aim is to apply the concept of automaticity to another complex process: ST (Tables 1, 2).

**Table 1:** Schema modes investigated in the present study.^1^

Schema mode	Definition
Vulnerable child	The patient believes that nobody will fulfill his needs and that everyone eventually abandons him. He mistrusts others and believes that they will abuse him. He feels worthless and expects rejection. He is ashamed of himself and he often feels excluded. He behaves like a small, vulnerable child that clings to the therapist for help, because he feels lonely and believes there is danger everywhere.
Detached self-soother	The patient seeks distraction in order not to feel negative emotions. He achieves this by soothing behavior (e.g. sleeping or substance abuse) or by self-stimulating activities (being fanatical or occupied with work, the internet, sport or sex).
Detached protector	The patient cuts off strong feelings because he believes that such feelings are dangerous and can get out of hand. He withdraws from social contacts and tries to cut off his feelings (sometimes this leads to dissociation). The patient feels empty, bored, and depersonalized.
Self-aggrandizer	The patient believes that is superior and entitled to special rights. He insists that he should be able to do or have what he wants, regardless of what others think. He shows off and denigrates others to augment his self-esteem
Demanding parent	The patient feels that he must fulfil rigid rules, norms, and values. He must be extremely efficient in meeting these. He believes that whatever he does is never good enough and that he must strive harder. Therefore, he pursues his highest standard until it is perfect, at the expense of rest and pleasure. He is also never satisfied with the result. These rules are also internalized by (one of) the parents.
Punitive parent	The patient is aggressive, intolerant, impatient, and unforgiving toward himself. He is always self-critical and feels guilty. He is ashamed of his mistakes and believes he had to be punished severely for them. This mode is a reflection of what (one of) the parents or other educators used to say to the patient in order to belittle or punish him.
Healthy adult	The patient has positive and neutralized thoughts and feelings about himself. He does things that are good for him and this leads to healthy relationships and activities. The Healthy Adult mode is not maladaptive.

^1^From van Vreeswijk et al. (33). Copyright 2012 by John Wiley & Sons. Reprinted with permission.

**Table 2:** Schemas investigated in the present study.^1^

Schema	Definition
Emotional deprivation	The patient expects that others will never or not adequately meet his primary emotional needs (e.g., for support, nurturance, empathy, and protection). He feels isolated and lonely.
Emotional inhibition	The patient inhibits emotions and impulses because he believes that any expression of feelings will harm others or lead to embarrassment, retaliation, or abandonment. He lacks spontaneity and stresses rationality.
Defectiveness/Shame	The patient believes that he is internally flawed and bad. If others get close, the will realize this and withdraw form the relationship. The feeling of being worthless often leads to a strong sense of shame.
Mistrust	The patient is convinced that others will intentionally abuse him in some way or that they will cheat or humiliate him. These feeling vary greatly and the patient is continuously on edge.

^1^From van Vreeswijk et al. (33). Copyright 2012 by John Wiley & Sons. Reprinted with permission.

Given the fact that habit theories of addiction are increasingly critiqued because there is growing evidence that drug use is more goal-driven than cue- or stimulus-driven [see (29)], we suggest that the ST model is a potentially valuable addiction theory that can function as an alternative to the ‘habit theories’ of addiction (6, 7, 34, 35). It is considered especially promising for patients with SUD and comorbid borderline personality disorder (e.g., 24). Further exploring ST in the context of addiction, and reflecting on how schema concepts apply to SUD therefore seems like a valuable venture. The framework of ST is comprehensive enough to conceptualize the complexities of automaticity and also does not regard addictive behavior as a merely stimulus-driven affair. It sees addictive behaviors as ways of coping with schemas, which can therefore be conceptualized as resulting from schema mode activity. Thus, rather than viewing substance abuse as a ‘habit’ that is stimulus-driven or cue-elicited [e.g., (6)], it regards substance abuse as a result of the automatic activation of schemas and the subsequent automatic adoption of schema modes (36). Furthermore, whereas habit theories suggest that this automaticity is the result of the strengthening of stimulus–response associations [e.g., (37, 38)], ST models suggest that automaticity is the result of an extensive learning history of adopting specific dysfunctional coping strategies in certain contexts (39). Finally, levels of explanation differ between the two theories: instead of using lasting brain changes as an explanation for the persistence of automatic substance use and the high relapse rates (e.g., 40), ST focuses on a psychological explanation for the difficulties to overcome substance abuse. These difficulties lie within the deep-rooted nature and the psychotraumatic origin of maladaptive schema modes (25).

Unravelling automaticity of the schema modes underlying addictive behaviors might help the treatment of addiction. Within this paper, we focus on the role of automaticity in schema modes and its role within different stages of ST, targeting addiction. Potentially, ideas on automaticity in addiction might increase the understanding of the mechanisms of ST (especially for addiction) and vice versa. In this, we will focus on severe addiction, which is often accompanied by personality disorders (41). We believe that the principles of automaticity in schema modes will be more pronounced if we direct our attention to severe pathology. We start with a brief ‘translation’ of the different automaticity criteria to schema modes. We then discuss the case of Jonathan, a patient with an alcohol use disorder and a narcissistic personality disorder and reflect on the automatic nature of Jonathan’s schema modes. We then analyze these reflections to identify specific patterns in each of the automaticity criteria and we explore questions that remain, followed by suggestions for clinicians and concrete suggestions for future research. The case of Jonathan is a hybrid case example (42), that the authors created for the present article. This case is constructed from actual ST cases that the first author encountered during his work as psychotherapist. Common themes from ST in patients with SUDs and personality disorders were highlighted, in order to vividly illustrate ST in this population and to underpin the theoretical claims that we make, while guaranteeing confidentiality.

### Schema modes and automaticity

When we apply the automaticity criteria put forward by Moors (17) on schema modes, we suggest the following. First, with regard to the consciousness-unconsciousness dimension [in the present article, we prefer to use the term ‘(self-)awareness’ instead of ‘consciousness’], because the latter is multi-interpretable (and often contrasted with being unconscious [asleep, or in a coma]), we consider that schema modes are automatic in the sense that they are unaware when a patient is unaware of the triggers (i.e., comparable to the ‘input’ described by Moors); and/or the emotional states of mind (schema modes; comparable to the ‘process’ described by Moors); and/or the behaviors that are a part of them (comparable to the ‘output’ described by Moors). Within ST, we suggest to add an additional component of awareness that relates to a deeper understanding: that a patient is not yet completely aware of a schema mode when he has not understood how and when the different components of a schema mode have developed and how they relate to each other. In other words, rather than an awareness of surface triggers, emotional states of mind and related behaviors, we suggest that there is full awareness when there is a historical understanding of the development of the schema (the ‘in-depth’ or historical trigger) and the schema mode (perhaps best called ‘self-awareness’).

With regard to the controlled/controllable-uncontrolled/uncontrollable dimension, we consider that a schema mode is automatic in the sense that it is uncontrolled or uncontrollable when a person does not have an intention to adopt a specific schema mode (e.g., a trigger automatically causes the activation of a specific schema and coping response, and the patient did not choose this and is not able to stop it); and/or they do have the intention to stop but they are not able to do so (e.g., a patient might have an intention to adopt the Healthy Adult mode but cannot achieve this because another mode takes over instead); and/or there is no causal relationship between the intention to attain a schema mode and being successful in attaining it (e.g., a patient might have the intention to adopt the Healthy Adult mode and indeed does not adopt the Vulnerable Child mode, but only because of external reasons: for instance, the trigger might have disappeared).

Third, considering the inefficient-efficient dimension, a schema mode can be regarded as automatic in the sense that it is efficient when a person does not require much cognitive capacity in order for the process of schema activation and coping to occur. This means that a schema mode occurs, regardless of the amount of cognitive resources that are available to the patient. When it comes to undesirable schema modes, we suggest that these actually have the largest chance to be adopted when cognitive resources are depleted. In contrast, the Healthy Adult mode generally requires more resources or effort, due to a lack of automaticity. Thus, when a patient is tired or highly emotional, unhealthy modes strike more often than when a patient is well-rested. In the latter case, the patient might be more able to adopt the Healthy Adult mode. It is possible to examine this automaticity dimension by examining the types of triggers that result in the activation of a schema mode. We suggest that a schema mode is highly efficient when it is triggered by a relatively weak trigger (e.g., being ignored in the street by a vague acquaintance) and less efficient when it is only triggered by strong triggers (e.g., being abandoned by a lover).

Fourth, with regard to the slow-fast dimension, we consider a schema mode as automatic in the sense that it is fast when it happens quickly: specific triggers immediately cause the activation of a schema, and the schema immediately triggers a coping style, without any time for reflection. In case of psychopathology, undesirable schema modes are often triggered more quickly than the Healthy Adult mode.

Because automaticity is considered to be dimensional and gradual, it is difficult to definitively state that a specific process or behavior happens completely automatically. However, in order to understand automatic behaviors and how to change them, it is important to reflect on automaticity in all its facets. Furthermore, reflecting on automaticity in a therapeutic setting can help therapists and patients to understand their progress. We suggest that there are three important therapeutic goals that relate to automaticity. One goal is to make dysfunctional schema modes less automatic: therapists strive to help patients to become increasingly aware of their schemas, the triggers of these schemas, the state of mind that comes with these schemas, their coping behavior, and the consequences of these behaviors. We also want patients to experience control. This means that they can choose not to engage in undesirable coping, or that when they do, they are able to reflect on it and to stop and choose to engage in more healthy behavior instead. Furthermore, we should strive to make a process less efficient in the sense that unhealthy coping is no longer effortless. We also should aim to make unhealthy coping less fast, leaving more time for processes to become conscious and controllable. This decrease of automaticity in schema mode activity enables the therapist to apply ST techniques that are aimed at trauma processing, like imagery rescripting and chair techniques.

Another therapeutic goal is to make healthy behavior more automatic in the sense that by the end of therapy, a client no longer needs to be constantly aware and vigilant on how their schemas and unhealthy coping modes are triggered, since they will automatically adopt the Healthy Adult mode. However, before a patient can reach this state, we must make the Healthy Adult mode *less* automatic: first, the patient needs to become aware of the Healthy Adult and needs to understand which components of the Healthy Adult are already present and functioning well, and which components are underdeveloped. Furthermore, the client needs to learn which requirements need to be met in order for the Healthy Adult to flourish. It also means that the client needs to exert control: to develop clear intentions to engage in Healthy Adult behavior, and to learn on how to act on these intentions. Furthermore, it means that initially, activating the Healthy Adult mode is a slow and effortful process. However, as therapy proceeds, this process becomes easier: this automaticity could even mean that the client, at some occasions, does no longer have to intentionally choose to engage in healthy behavior, and that this happens quickly and effortlessly instead.

A third goal regards the automaticity of the Vulnerable Child. Whereas the Vulnerable Child is not associated with the functional behavior of the Healthy Adult, it cannot be lumped together with the dysfunctional coping modes either. The Vulnerable Child provides the therapist with useful information about the patient’s needs and it is necessary to access the Vulnerable Child to reach full (historical) awareness of the schema modes. Just like with the other schema modes, the first step is to try to make the mode less automatic in the sense that it needs to become more aware, less efficient and less fast. However, when it comes to the controllability of the Vulnerable Child, the goals differ: whereas the Vulnerable Child is usually controlled (e.g., inhibited or actively fought) by dysfunctional coping modes, the goal is to ‘free’ the Vulnerable Child from this control. This freedom will then result in a gradual fading out of the intensity of the Vulnerable Child, often through trauma processing. Eventually, the Healthy Adult mode is able to exert control over the Vulnerable Child mode by reassuring and comforting it, when triggered.

We suggest the ST process can roughly be divided into three parts: the start, middle, and end. On the basis of the case of Jonathan, we examine for each phase of the ST process to what extent different features of automaticity are present and how they change over time.

## The case of Jonathan

### Prelude

Jonathan hates losers. He makes sures that I am fully aware of this, by mentioning it twice in the first 10 min of our first ST session. Apart from making me a little nervous (does he classify me as a loser?), he intrigues me. What is the use, the function, of this unusual communication?

In our sessions in the weeks that follow, I learn that Jonathan is an amiable man, who behaves kinglike. His posture, stride, and gestures exude confidence. He treats me kindly (as long as I do not talk too much), and tells me at length about his life. In short: he owned a major company that sold kitchens, worked hard, earned a lot of money, took excessive financial risks, and went bankrupt. He lost his fortune, his two houses, his Jaguar and, eventually, his marriage. Throughout his adulthood he drank alcohol regularly. When he turned 40, his whisky drinking became a daily habit (he is now 51), helping him to cope with the stress caused by him leading his company, and the monstrous working hours that accompanied it. Last year, when his life fell apart, he became homeless, after an alcohol intoxicated fight with his wife. He lived in his car, drinking 24/7, and, eventually, found himself on an abandoned car park, with the barrel of a hunting rifle in his mouth. He would have been dead, if not for the fact that he had to vomit at that very moment. Realizing the absurdity of his situation, he decided to reach out for help. Jonathan was hospitalized in an addiction clinic, and soon regained some of his old strength. He ate and slept well in the clinic, remained abstinent, helped other patients, and was capable of finding a place to stay after his discharge, helped by his intelligence and business skills. He entered a 3-year program that helped him to pay off his debts. At Antes, a large mental health care organization in Rotterdam (Netherlands) he started outpatient cognitive behavioral therapy for his alcohol use disorder. This treatment was a painstaking process, as Jonathan refused to take any advice from his therapist. He ignored homework assignments and became patronizing and devaluating when his therapist tried to prevent Jonathan from declaiming his monologues. However, he remained sober. In a psychological assessment, a narcissistic personality disorder was classified. Thereupon, he was referred to ST.

### Start

I learn very soon that Jonathan is easily offended. The only way to connect with him emotionally during the first phase of our therapy, is to validate the painfulness of his loss of his prosperity and prestige. The emotional pain that this has caused, and the vague feeling of emptiness and loneliness that will not go away form his motivation for therapy. With a lot of effort on my behalf, and very slowly, we make a case conceptualization (including a mode model, according to ST). I feel that there is a lot of information that Jonathan is not sharing, but our rudimentary model seems sufficient for the time being. We conceptualize a Self-Aggrandizer mode and a Detached Self-Soother mode, that together shield the painful dynamics between the Demanding Parent mode and the Vulnerable Child mode (which we call ‘Jonathan’, in order to avoid his connotations with inferiority regarding the term Vulnerable Child). The Demanding Parent mode tells the Vulnerable Child mode not to experience any ‘weak’ feelings, because these feeling lead nowhere (schema of Emotional Inhibition). Instead, he has to work hard and strive for success, clearly echoing the beliefs of Jonathan’s father during Jonathan’s early years. Father instructed his son to be a ‘real man’ and to avoid weakness. Jonathan’s mother was not there for him unconditionally either: she suffered from an undisclosed somatic condition and spent much time in bed. The behavior of his mother added to the idea that his emotions were irrelevant and not of interest to others (schema of Emotional Deprivation). While this picture of Jonathan’s childhood emerges in therapy, he starts to get a notion of the function of his coping behavior. But it is only in the middle part of the therapy that he starts seeing the dysfunctional nature of this behavior. Working hard and drinking alcohol (Detached Self-Soother) helps him to avoid his emotions (and negative messages about these emotions stemming from the Demanding Parent mode). Depicting himself as a dominant, successful man (Self-Aggrandizer) helps him to be the kind of man his Demanding Parent mode wants him to be. In this I have the idea that I do not get the complete picture, but my questions focusing on other schemas than Emotional Deprivation and Emotional Inhibition (e.g., Shame/Defectiveness) or inquiring the existence of a Punitive Parent mode lead nowhere. The overall increase of insight seems to cause a rise in suffering.

In other words, therapeutic gain is accomplished in that I (with help of our mode model) am allowed to not only validate Jonathan’s pain caused by his recent societal adversity, but also may validate the function of his dysfunctional coping modes. Nevertheless, the ‘charade’ of chair work that I try to do, faces strong resistance. Most notably, me trying to address the mode of the Vulnerable Child, seems to fill him with disgust, especially when I direct my attention on the neglect that he has experienced in his childhood. In other words, historical validation of the feelings of the mode of the Vulnerable Child appears to be very difficult. Whenever I try to do this, Jonathan obviously feels patronized and often suddenly starts to devaluate (me). ‘Perhaps you provide therapy to junkies who buy it when they are addressed like 10-year-old. But I do not… Nevertheless, I can see where you are coming from: although I do not do drugs, I’m a junkie too.’

### Start phase – reflection on automaticity

In this phase, Jonathan’s modes are highly automatic. During this phase, however, some differentiation occurs regarding the level of automaticity.

#### Awareness

Jonathan enters therapy with a vague notion of the existence of the Vulnerable Child mode: the pain he feels stemming from the loss of his wealth, the vague feeling of emptiness. In this phase, he also starts to become aware of being in the Vulnerable Child mode and in what types of behavior this results (e.g., when he and his therapist discuss the end of Jonathan’s marriage, he is conscious of becoming sad and of his tendency to stop talking). He is also able to identify direct, superficial triggers of his Vulnerable Child mode (e.g., the therapist talking ‘too much’ makes him uneasy). Even though he seems to be aware of these important components, this awareness remains superficial: a deeper self-awareness of the schemas that are involved in the Vulnerable Child mode and how these schemas have developed in the past is still lacking. Regarding the (dys)functioning of coping modes (Detached Self-Soother, Self-Aggrandizer), he realizes when he detaches and when he acts in as if he is superior, but sees this as normal and rightful behavior that might be triggered by financial problems (Detached Self-Soother) or by a therapist who assigns homework (Self-Aggrandizer). When the therapist tries to explore the nature of the parent modes, she encounters the Demanding Parent mode, telling Jonathan not to be weak and work hard. He becomes aware of the functioning of the Demanding Parent mode, but, again, is unaware of the dysfunctional nature of this schema mode. The therapist suspects the existence of a Punitive Parent mode, but it remains out of sight – it is possibly operating fully unawarely. In this start phase, intensive work is done to help Jonathan to become more aware of the existence and the functioning (i.e., when are they activated, and what are their causes and consequences?) of his schema modes. At the end of the start phase, he is more or less aware of the functioning of his Vulnerable Child mode, his coping modes and his Demanding Parent mode. He is quite unaware of the detrimental functioning of the coping modes and the Demanding Parent mode. Further, the ‘historical understanding’ of his schema modes (understanding how his childhood full of emotional neglect made him develop these schema modes) is absent. This is hypothetically due to fear of fully grasping the extent of his emotional pain. Some parts of Jonathan’s Healthy Adult mode are well developed; e.g. he has been able to start repaying his debts, find a place to stay, find a therapist etc. Other parts of his Healthy Adult are completely underdeveloped, like healthy self-soothing, being mild, connecting emotionally to others. Jonathan is largely unaware of the presence and the functioning of the Healthy Adult mode in him. In therapy, the ‘good parenting’ part [being mild, connecting to others etc.; (43)] of the Healthy Adult mode is performed by the therapist. Probably, the foundation of the development of this part of the Healthy Adult mode is installed through experiential (instead of cognitive) learning, hypothetically also to avoid pseudo Healthy Adult behavior. In the start phase, there is hardly any change in awareness regarding the Healthy Adult mode.

#### Control

Jonathan has little control over the functioning of his Vulnerable Child mode, coping modes, Parent modes and Healthy Adult mode. In this phase, Jonathan becomes quite aware of the appearance of the Vulnerable Child mode. He has no control over this schema mode, but does not need to control it: here, his coping modes (Self-Aggrandizer, Detached Self-Soother) take over which block the occurrence of the Vulnerable Child mode. He lacks real control over this schema mode.

He seems to exhibit some control over his Self-Soother mode, in that he does not drink any alcohol during this phase. However, it is difficult to say whether there is a causal link between the two: Jonathan is indeed able to control his drinking behavior, but this may not be due to a conscious decision not to engage in behaviors related to the Detached Self-Soother. Rather, his sobriety is possibly better explained by the automatic (uncontrolled) functioning of an aspect of his Healthy Adult mode that has been well-developed: his impressive willpower. Another possibility is that Jonathan’s sobriety is induced by the functioning of the Demanding Parent mode, who tells him to be a model patient.

Regarding the Self-Aggrandizer mode, there is no control whatsoever: Jonathan does not have any intention to abstain from behaving in a superior or derogatory way and thus fully engages in the behaviors that are associated with it. The same applies to the Demanding Parent mode: Jonathan has no intention to change his strict attitude regarding his emotions. The (alleged) Punitive Parent remains uncontrolled, since it operates completely outside of awareness.

#### Efficiency

In this start phase, the activation of all schema modes is highly efficient. Regardless of Jonathan’s efforts to suppress the Vulnerable Child, it still enters the scene (and often for short moments, due to the activation of coping modes), when Jonathan’s recent adversities are discussed. Effortlessly, the Demanding Parent mode becomes active when the therapist tries to comfort the Vulnerable Child Mode. And equally effortless, Jonathan’s Self-Aggrandizer mode starts devaluating the therapist when Jonathan feels the pain caused by the dynamics between the Vulnerable Child mode and the Demanding Parent mode. The facets of the Healthy Adult mode that are developed operate efficiently as well: Jonathan regaining control over his financial situation is obvious to him.

#### Speed

Speed and efficiency in schema modes seem to be strongly related. The occurrence of various schema modes happens fast, often surprising the therapist. Triggers immediately result in the activation of a schema mode, including accompanying behavior. The triggering of one schema mode can lead to the triggering of another mode, which can lead to the appearance of third schema mode, all in a very brief period. This often confuses the therapist. For example, validation of the feelings of the Vulnerable Child mode by the therapist often instantaneously lead to activation of the Self-Aggrandizer mode. On some occasions, this leads to the activation of the Demanding (Punitive?) Parent mode. This can be seen in the above-described example, in which the therapist has just started to validate the feelings of the Vulnerable Child, and Jonathan reacts: ‘Perhaps you provide therapy to junkies who buy it when they are addressed like 10-year-old. But I do not (Self-Aggrandizer). …nevertheless, I can see where you are coming from: although I do not do drugs, I’m a junkie too (Parent mode).’ This so-called mode flipping (44) is often caused by interaction between Jonathan and the therapist.

### Middle

After extensive explanation and reassurance, Jonathan agrees to do Imagery Rescripting. These Imagery Rescripting leads to an acceleration of the therapy. Jonathan is no longer capable of holding back his emotions. Very often he is deeply saddened and upset by the childhood memories that appear. He cries intensely and needs a lot of comfort from me. Mode flips during and after such imagery sessions appear, in which he becomes mad at me and starts devaluating or even insulting me. It seems like his Self-Aggrandizer mode needs to end the pain coming from childhood memories and the fear that is triggered by my closeness when I’m comforting him. At other moments, his Punitive Parent mode targets his Vulnerable Child mode and encourages him to kill himself. At various moments I have to intervene in between sessions because of suicidality. In an ultimate low the Detached Self-Soother takes over and Jonathan starts drinking whisky again, something that he promised himself never to do again. The drinking becomes unmanageable once more and admission to a detox facility is unavoidable.

All this hardship discloses interesting information. Jonathan has previously been too ashamed to tell me that he was systematically bullied during primary school. In Imagery Rescripting, he starts sharing painful memories on his years of victimization. He got insulted, spit at, threatened, beaten, and sexually assaulted by classmates for several years. His parents did not support him in any way: his mother looked the other way, his father laughed at Jonathan’s tears and told him to ‘man up’. Because of this, he is suffering from the schemas Shame/Defectiveness and Mistrust. This knowledge helps me to specifically address these schemas by being as trustworthy as I possibly can (e.g., in answering emails, keeping promises, making sure that our appointments have a solid place in my agenda, protecting privacy). Further, I value and praise every authentic part of him that I am allowed to see. The hypothesized Punitive Parent mode appears to actually be a part of Jonathan, closely tied to his Shame/Defectiveness schema (due to the bullying in his childhood).

One session, when I try to comfort him, he automatically switches to an attacking mode (Self-Aggrandizer, or perhaps more likely Bully and Attack). He states that I’m wrong when I refer to his tears: he is not crying. ‘Obviously, these glasses do not suffice, Four Eyes!’ He hurts my feelings, but fortunately not so bad that I am prevented from empathically confronting him. He is shocked when he sees that I am really hurt by his behavior. Talking this through, Jonathan is able to make major progress in understanding his own dynamics. When someone comes close, he gets scared and devaluates or lashes out. I have been trying to explain this to him before, but this experiential learning is much more powerful. His realization makes him decide to change this behavior. He actually starts changing it by inhibiting himself whenever he feels the urge, or, in cases when he is unable to refrain himself and devaluates someone, apologizing for it. His emotional pain remains prominent. He feels it even more strongly, since it is not covered up anymore by his dysfunctional coping modes.

Even minor, unintended offenses (me being 5 min late for a session, his favorite bread being sold out at the bakery), still upset him. But Jonathan holds on. More and more he ‘sees’ what happens, and tries to calm himself. In this, he is trying to copy my behavior. He becomes aware of what the use of a Healthy Adult mode might be. He uses audio clips that we have made in therapy, or looks at a picture of himself as a child. Jonathan even goes on a date, stemming from a strong wish to heal himself. This is not successful; he leaves the restaurant early. It feels so vulnerable to be with an attractive woman, without bragging and impressing her!

### Middle phase – reflection on automaticity

During this phase, Jonathan’s modes are becoming less and less automatic.

#### Awareness

The awareness of the functioning of the various schema modes further increases. Jonathan’s awareness of the Vulnerable Child was already relatively advanced, since he was aware of the mode itself, the triggers, and the resulting behaviors. However, in this stage he gains a deeper awareness of the scale of his emotional pain. He becomes capable of linking his present pain with his childhood suffering, understanding the origin of the schemas that drive the Vulnerable Child. The application of Imagery Rescripting helps him to feel his sadness and shame, linked to traumas. Further, patient and therapist learn that the Demanding Parent mode is accompanied by the Punitive Parent mode, capitalizing on a grave sense of inferiority. Jonathan begins to understand that the negative messages of his childhood bullies have been internalized in his Punititive Parent mode. His awareness of the activity of the Punitive Parent fluctuates, but the trend is upward during this phase. At moments, he merges with the Punitive Parent mode, unaware of its pathologic nature. In other instances, he appears to be fully aware of this schema mode (including its historical roots). At first, Jonathan is unable to allow the therapist to comfort and support him. Step by step, and as a result of the deeper understanding and awareness regarding the Vulnerable Child, Jonathan starts to feel empathy for this Vulnerable Child and grants access to it. He learns to trust the therapist, resulting in a child-like dependence, seeking a lot of reassurance in between therapy sessions. Another milestone in awareness of schema modes occurs when Jonathan offends and hurts the therapist. This shocking moment goes beyond a superficial awareness. It helps Jonathan understand how his Self-Aggrandizer (or perhaps Bully and Attack) mode gets activated when he feels vulnerable. He understands historically: he prevents being bullied by bullying others. This understanding is not just cognitive; Jonathan’s understanding obtains an emotional component as well. He starts to understand his schema modes emotionally, or “embodied” (45).

Regarding the Healthy Adult mode, Jonathan becomes more and more aware of the requirements for the Healthy Adult to appear (e.g., he needs to be well-rested, engage in healthy leisure activities), he learns to see and appreciate when he is in the Healthy Adult mode, and he can identify healthy behaviors. He also learns about the underdeveloped components of the Healthy Adult., like self-soothing, connecting with others, trusting them etc. These latter components he learns by being in a therapeutic relationship. He learns to trust his therapist and finds out that being comforted can feel good (and awkward at the same time).

#### Control

In the middle phase of the ST, Jonathan experiences very limited control over his schema modes. Because he now becomes, at least to a considerable extent, aware of all his schema modes, he is able to develop clear intentions about inhibiting certain modes (e.g., the Self-Aggrandizer; Detached Self-Soother) while activating others (e.g., the Healthy Adult). However, regardless of his intentions, it is very difficult to engage in the desired Healthy Adult mode: Jonathan suffers from deep, uncontrollable pain (Vulnerable Child mode), often leading to suicidality (inflicted by the Punitive Parent mode). Because the coping modes start to have less power over the Vulnerable Child mode, it becomes so active, uncontrollable, and threatening, that the Detached Self-Soother mode takes over again, and Jonathan, unwillingly, starts to drink again.

The lack of control over the functioning of schema modes paired with the increase of awareness of the functioning of the modes, might explain the increase of suffering, so often seen in the case conceptualization phase in ST (24, 46).

Important in this phase is the empathic confrontation when Jonathan applies the Bully and Attack mode toward the therapist. On the basis of a strong therapeutic relationship, Jonathan is shocked by the consequence of the behavior coming from his Self-Aggrandizer mode. He decides to take responsibility for his actions: his Heathy Adult mode is subject to a growth spurt. He starts being able to inhibit his tendency to devaluate others (a growing control over his Self-Aggrandizer mode), and in cases were the inhibition falls short, he apologizes (Healthy Adult mode). When Jonathan goes on a date, he stills lacks control over the Vulnerable Child and Punitive Parent modes. He is unable to address the Punitive Parent mode effectively and to apply the Healthy Adult mode to calm down his Vulnerable Child mode.

#### Efficiency

In the middle phase of ST, the efficiency of the functioning of the Vulnerable Child mode, parent modes and dysfunctional coping modes is still very high, although some decrease in this component of automaticity is observed. Discussing or targeting painful childhood memories very easily leads to strong emotions (Vulnerable Child mode). After vulnerability in a therapy session, often an effortless mode flip to the Punitive Parent mode occurs, leading to suicidality. And emotional intimacy between therapist and patient leads smoothly to the activity of the Self-Aggrandizer mode. The above-mentioned empathic confrontation after the devaluation of the therapist helps to decrease the efficiency of Jonathan’s modes. This empathic confrontation must not be seen as some kind of epiphany, but rather as tipping point, as a climax of all the work that was done up to this moment in therapy. The activation of the Vulnerable Child mode and the Punitive Parent mode decrease in efficiency, especially with relatively minor triggers. When, for example, the therapist is late for a session, Jonathan’s Defectiveness/Shame schema is still easily triggered (input). However, especially the behavior (output) belonging to the Vulnerable Child mode and the Punitive Parent mode become less efficient. Although he gets upset by these minor incidents, the schema triggering does not lead automatically to dysfunctional behavior. The activation of the Self-Aggrandizer mode decreases even more in smoothness. It seems like Jonathan is startled every time his Self-Aggrandizer is activated, since the moment he hurt his therapist, disrupting efficiency. Jonathan’s Healthy Adult mode starts to develop more fully during this phase. The inhibition of dysfunctional behavior by the Healthy Adult mode takes a lot of effort and deliberate choice.

#### Speed

Although Jonathan’s mode-flips show that this part of the automaticity of his schema modes is still very high as it regards speed, some slowing down seems to appear. He tries to inhibit his dysfunctional coping modes, and to behave more healthily. This process comes about in fits and starts.

### End

After two-and-a-half year of ST, Jonathan and I conclude that his sadness, anger and pain have substantially decreased. His craving for alcohol is slowly diminishing during this phase. Whenever he senses craving, for example when seeing a commercial of a beer brand, he is able to cope in an adaptive way (calling a friend, going for a walk with his dog). He is, however, somewhat disappointed by the fact that living this new life costs so much energy. He has hoped that making Healthy Adult mode choices would have become more natural. Every time one of his schemas (Emotional Inhibition, Emotional Deprivation Shame/Defectiveness, Mistrust) is triggered, he has to work so hard not to get subtly devaluating, or rush into some unhealthy self-soothing activity. In other words, the healing of the scars of the Vulnerable child seems to have a head start on the change of the dysfunctional coping modes. We decide to continue our therapy. I suggest that practicing his new behavior intensively will help it to become more automatic.

For another year we meet up in bi-weekly sessions. Because of his high levels of energy, it is easy to motivate Jonathan to engage in all kinds of activities. He starts volunteering at a pet shelter, cooks meals for his neighbors, regains contact with his ex-wife, and starts an internet-based company, selling batteries. It is not hard to see his narcissism shine through in his new activities (he even becomes manager of the shelter). We discuss this regularly, laugh about it, think about the risks. But I also stress the strengths of his high energy and dominance, as long as they do not prevent his basic needs (connection, safety) from being fulfilled.

After a long therapy break, due to the holidays, Jonathan and I meet again. While walking together to my office, I am once again struck by his confident stride and the strength that he projects. In my office he tells me kindly about all his experiences in the last months. I just listen, there is no reason to interrupt. Hearing his stories, I begin to realize that Jonathan’s healthy behavior has become more effortless. When I tell him this, he smiles. He is already aware of it.

### End phase – reflection on automaticity

During this phase, Jonathan’s child modes, dysfunctional coping modes, and parent modes show a strong decrease in automaticity. Some automaticity develops in the functioning of his Healthy Adult mode.

#### Awareness

In this phase Jonathan is very well aware of the functioning of the Vulnerable Child, the Detached Self-Soother, the Self-Aggrandizer (and his tendency to bully when the pressure gets high), the Demanding Parent and the Punitive Parent. This awareness has now deepened to a full understanding of the surface and historical triggering of schemas (input), consciousness of the state he comes into (process) and the dysfunctional behavior that he tends to adapt (output). This self-awareness leads to a rather steady state wherein he does not fully identify with the different maladaptive schema modes anymore. Furthermore, understanding the way in which his schemas have developed has created a sense of empathy towards his Vulnerable Child. When Jonathan listens to its needs, he is no longer going through the motions, rather, it stems from a desire to comfort the Vulnerable Child. Concurrently, Jonathan does identify with the Healthy Adult mode. This is further depicted by his capability to laugh about his own narcissism. The therapist and Jonathan become aware of another element of the functioning of the Healthy Adult mode, with regard to his alcohol use disorder. Previously, Jonathan denied experiencing craving for alcohol. His relative steadiness in remaining sober might have been the consequence of him trying to be a perfect patient for his therapist (based on the Demanding Parent mode). He now feels free to experience craving and talk about it, thus enabling meeting the needs of the Vulnerable Child mode.

#### Control

The amount of control over the schema modes increases significantly in this phase. Probably strongly facilitated by trauma healing, Jonathan no longer merely intends to control the activity of the Vulnerable Child by inhibiting it, but he learns that, paradoxically, it is more adaptive to express the feelings and thoughts of the Vulnerable Child mode. In others words: controlling by letting go. Jonathan has learned to believe that he is a valuable human being and that most others are trustworthy. Schema triggering does not overwhelm him anymore, adding to the ‘controllability’ of the Vulnerable Child mode. He is capable of directing his Healthy Adult mode: when he starts craving for alcohol, he is able to fulfil his unmet need for attachment and calls a friend or connects with his dog. In this, the Healthy Adult mode seems to exercise control over the Self-Soother mode. Jonathan’s Healthy Adult mode is further capable of countering the activation of his Punitive Parent mode. He copies the forceful limitation of the Punitive Parent by the therapist. Jonathan’s limiting of his Punitive Parent mode is also facilitated by his empathy with the Vulnerable Child mode and the related growth of his self-esteem. He believes that, for example, it is untrue that he has to commit suicide whenever things do not go according to plan.

#### Efficiency

In this therapy phase, Jonathan’s emotional wounds are healed, and, although the scars are visible, his emotional arousal is far less pronounced whenever a schema is triggered. In other words, the Vulnerable Child mode still gets triggered, but the emotional state that accompanies this is less intense, and the corresponding behavior (e.g., clinging to the therapist in despair) has disappeared. The residual pain that is triggered, is manageable for the Healthy Adult mode. In this end phase, Jonathan is bothered most by the fact that his new lifestyle costs so much energy. For almost 15 years he has guarded his pain by soothing himself in a dysfunctional way and by keeping others at a distance. Despite his progress, the structure of Jonathan’s coping modes (the Self-Soother mode and the Self-Aggrandizer mode) is, however, largely standing. It costs him lots of effort to not relapse in these schema modes (working hard, trying hard to excel. In this, a confusion between the Healthy Adult and the Demanding Parent is lurking). It is only in investing intensively in social activities (outside the therapy) that Jonathan is able to strongly diminish the efficiency of his maladaptive coping modes, and, even more importantly, increase the efficiency of his Healthy Adult mode. The efficiency of Jonathan’s Punitive Parent mode declines more rapidly than the efficiency of his maladaptive coping modes. He has become convinced that he is not worthless, which helps him to counter negative thoughts often right away.

#### Speed

in this phase of therapy too, the speed of the functioning of schema modes seems highly related to their efficiency. The activation of schema modes, especially in emotionally charged circumstances, is still rather fast (input). However, the activation of the emotional state of the schema mode (process) and the coping behavior (output) has substantially slowed down. For example, when Jonathan is in a sad mood, and whilst being in this state he is exposed to a beer commercial, the Detached Self-Soother mode is activated and craving is swiftly triggered. However, the ties between the sad mood and his Emotional Deprivation schema are far less strong, slowing down the process. The activation of the schema mode behavior (panicking, searching for a quick fix like alcohol) has slowed down even more, enabling the adoption of the Healthy Adult mode.

## Discussion

Our analysis shows that it is helpful and important to apply the componential view on automaticity (17) to schema modes and the way they change during the course of ST for patients suffering from substance use disorders and co-morbid personality disorders. First, we see that the initial changes in automaticity that a patient experiences are related to awareness. Whereas this awareness remains superficial in the start phase of ST (e.g., an awareness of the existence and occurrence of different schema modes and the accompanying behaviors), it deepens in the next stages, and develops into an awareness of relationships between schema modes, the behaviors that are a part of these schema modes, and the historical development of schemas and schema modes. In other words, the client develops a deeper understanding of their problems. This deeper understanding leads to more emotional suffering, opening the door to trauma-focused interventions. These interventions decrease the emotional arousal caused by schemas that become activated. Somewhat later in ST, patients develop an awareness of the Healthy Adult mode and the behaviors that are incorporated in this schema mode. Second, we see that following the changes in awareness and the strength of schemas, changes in controllability occur: the patient’s awareness of their schema modes (and triggers and consequences) allows them to form intentions not to engage in dysfunctional behaviors, and as the therapeutic process continues, they are increasingly able to fulfil these intentions. Slowly, the Healthy Adult takes over. Third, we see that following the increased awareness and controllability, changes in efficiency start to occur later in the process: although patients obtain deeper insight in the causes of their emotional wounds and their coping, it remains an effortful endeavor not to adopt unhealthy schema modes. It takes a lot of practicing (in daily life) to become able to adopt healthy behavior more effortlessly. Fourth, we see that whereas the triggering of schemas can remain very fast, the impact of this triggering is not as large as it was in the beginning: it does not automatically lead to dysfunctional behavior. Towards the end of ST, schemas are still swiftly triggered, but schema modes (and especially the behavioral part of schema modes) do not emerge that easily.

### Implications for schema theory

The application of a componential outlook on automaticity in ST has clear implications for the theory of ST. Schema theory is a coherent whole of concepts regarding psychopathology (e.g., basic needs, schemas, schema modes) and ideas about how to resolve this pathology (e.g., limited reparenting, experiential techniques, cognitive techniques). A thorough understanding of automaticity adds another layer to ST, a further understanding of the persistence of schema modes and mechanisms of change. First, schema modes are believed to be long-lasting, resistant to change and acting as self-fulfilling prophecies. This resistance to change might be understood in terms of automaticity. Automaticity researchers have already established that automatic behavior is the result of extensive learning, and that once a behavior is considered as automatic, it is extremely difficult to change [e.g., (47, 48)]. Whereas most literature on the way in which we learn automatic behaviors focuses on the learning of (co)occurrences of stimuli or stimulus properties (49), language learning [e.g., (50)], habit learning [e.g., (51, 52)], or the learning of motor skills [e.g., (53)], we suggest that similar processes are at play when schemas and schema modes are learned. Schema modes develop early in life and are therefore often out of awareness ever since they were developed. Due to the pervasiveness and persistence of the detrimental childhood circumstances, the schema mode (and the coping behavior that is a part of it) is learned extensively. Moreover, this behavior is consistently reinforced (i.e., the coping behavior provides a short-term solution to deal with the detrimental circumstances), strengthening the automaticity even more (54). The automaticity can further be strengthened by the fact that the behaviors occur under stressful circumstances (55).

Second, our exploration of the role of automaticity in ST also deepens our understanding of the therapy process. We suggest that ST follows a fixed process of de-automatization (through an increase in awareness, which enables trauma-focused intervention, followed by a gain in control; all this based on a general slowing down). The last phase of a successful therapy consists of automatization of the functioning of the Healthy Adult mode (with an increase in efficiency as most important part).

### Clinical implications

Our analysis has important implications for clinical practice. Awareness and controllability change subsequentially: first awareness and then controllability. This order causes a difficult and risky phase in therapy, in which awareness has grown, but control is largely lacking. First, awareness seems to be crucial for any change to occur. However, merely being aware of the input, the process, and the output is not enough to experience real therapeutic change. Gradually, during therapy, a deeper awareness of underlying mechanisms (the development of schemas and their historical functions) is necessary for lasting changes to occur. This understanding is not merely cognitive: it is emotional as well. Through increased awareness of the depth and magnitude of their problems, the emotional suffering of patients in ST rises substantially during this phase (especially when they have just reached sobriety, addictive substances often serving as means to feel less). But their lack of control prevents them from decreasing their detrimental behavior (which generates a substantial part of their suffering). This makes them at risk for relapse and drop-out. It is important to inform patients early in therapy about the possible occurrence of this state and support them maximally during it. This order of change (first awareness and later controllability) seems to be inherent, a necessity in ST. Therapists need to bear and accept the lack of control of their patients over their pathological behavior, and not press too hard on behavioral change in this phase. Awareness, emotional understanding of their own case and subsequent trauma-focused techniques are necessary before control develops. Furthermore, we learned that control goes beyond an individual having the intention and the ability to stop a particular behavior. It is also important to examine the underlying mechanisms: does the desired behavior (e.g., abstaining from alcohol) occur because the patient has control over their dysfunctional schemas? Or is it actually the result of another dysfunctional schema mode? In order for lasting changes to occur, we need to be sure that control is exerted due to adoption of the Healthy Adult mode. Control based on functioning of, for example, the Demanding Parent mode is hypothetically not leading to healthy, lasting change (‘repression’ might be a more appropriate term than ‘control’ here).

Both awareness and control thus seem be particularly informative to examine within the therapeutic process: for therapists, focusing on awareness is the first priority in ST. In ST, this involves taking time, in joint effort with a patient, in order to make a strong case conceptualization and a mode model, is very important. Further, it is important to regularly adjust this conceptualization based on new insights and to share these insights with the patient.

Whereas the therapist can clearly see drastic changes in awareness and controllability, the latter two criteria, efficiency and speed, show less apparent, more gradual changes during the course of therapy. Nevertheless, they are useful to keep in mind during the therapeutic process as they are indicators of (the durability of) changes. In a successful ST, a general slowing down appears, in which the functioning of al schema modes becomes less efficient. The activation of coping modes becomes less effortless. Mode flipping decreases, enabling limited reparenting of the Vulnerable Child mode. Even the functioning of the existing parts of the Healthy Adult mode slows down, as therapist and patient question the nature of the Healthy Adult mode (e.g., might the Demanding Parent mode be involved?). The last part of ST is used to enable the Healthy Adult mode to function more efficiently and quickly. Thus, reducing speed in the functioning of the schema modes is important. This can be done through zooming in on in-session schema mode activity in a calm, accepting way. The last part of ST should be dedicated to automatizing Healthy Adult behaviors. Often, it is only through intensive training that Healthy Adult behaviors are performed with less effort, automatically. This decrease in effort increases the likelihood that these behaviors will be executed in the future.

A last clinical implication of the focus on automaticity in schema modes might be that it can help us understand stagnation in ST. This stagnation may be seen as a stagnation in increase of self-awareness, increase of controllability, decrease of speed, or a decrease of efficiency in coping or parent modes. Instead, it might be regarded as a problem in building automaticity in the functioning of the Healthy Adult mode. Stagnation in the therapeutic progress regarding schema modes during ST might be based on the strength of the underlying schemas that drive these schema modes (i.e., schemas are hard to change). Hypothetically (39), behaviors that perpetuate schemas are reinforced by short-term relief, survival, and/or the human strive for equilibrium/the self-preservative nature of schemas (27). In other words, stagnation in ST possibly stems from the deeply-rooted (dys)functionality of schemas and the discomfort that comes with change. Knowing which element of automaticity stagnates, might be helpful guide in the application of suitable therapeutic interventions. For example, ST is sometimes concluded after patients have gained deep insight and when their traumas are healed. However, if they keep relapsing in alcohol use, it might be fruitful to consider intensive, practical training of inhibition of maladaptive coping modes afterwards. Although further research is needed to understand the value of automaticity for stagnation in ST, we suggest the following. When confronted with stagnation in awareness, return to the case conceptualization with the patient, and figure out whether it is accurate and adjust if needed. Other ways of increasing awareness of automatic behaviors might be through adding interventions that are focused on bodily sensations (stemming, for example, from body-oriented psychotherapy). Second, when control fails to develop, consider additional trauma processing techniques. Stagnation in efficiency of healthy behavior might ask for more hands-on practice. And last, a prolonged lack of slowing down in the patient (enduring mode flipping) asks for slowing down in therapy, for example through adding elements of mindfulness (56, 57). It is likely that mode flipping is related to fear of patients to be in the Vulnerable Child mode for longer periods of time and the expected despair that comes with it. Therefore, it seems advisable to introduce mindfulness in a graduated fashion (at first only in-session), in order to help patients get used to mindfulness. An alternative might be the application of basic CBT elements in helping patients to slow down (monitoring and naming emotions and automatic thoughts). The de-automatization of coping modes and parent modes and the automatization of the Healthy Adult mode seem strongly related to the concept of mindfulness, or meta-awareness, as it is conceptualized by Edwards (58). He suggests that training in mindfulness supports meta-awareness, through disidentification: ‘the ability to step back from a part of the self’ (p. 4). This stepping back helps recognizing that this part is not the whole self, and helps increasing self-control regarding automatic responses.

Looking at the clinical implications described above, one might argue that most suggestions made might already be part of good clinical ST practice (e.g., making a strong case conceptualization, focusing on slowing down). What might be the added value of a focus on automaticity in ST? Explaining the automatic nature of schema modes to patients helps them to be compassionate for the pace of their changes (‘changing automaticity takes time and hard work’) and to be hopeful. Further, schema therapists might feel supported by the idea that are addressing automaticity. In addressing automaticity, ST can be roughly divided into different phases: working on self-awareness, focusing on trauma, helping in gaining control, and focusing on automatizing the Healthy Adult mode. This awareness of phases helps therapists to stay on track and predict behavior. It also allows therapists to anticipate possible set-backs and to swiftly take appropriate action. Lastly, applying the concept of automaticity (as conceptualized in addiction) on schema modes helps, vice versa, to understand addictive behaviors as schema mode activity. This stresses the idea that addiction is not merely a brain disease asking for psychotropic medication and self-control interventions. Addiction therapy falls within the realm of psychotherapy too, and understanding of schema modes can help to demystify the automatic processes in addictive behaviors (‘the brain takes over’).

### Future research

In order to be able to fully use automaticity criteria as sources of information in clinical ST settings, it is important to further examine this through both quantitative and qualitative research.

First, it is important to conduct more qualitative research. It is important to note that our analysis concerns a case in which specific schema modes were active. Patterns in automaticity might differ depending on the constellation of schema modes that are at play. For instance, Jonathan’s coping modes tried hard to control his Vulnerable Child, meaning that he actually needed to give up some control in order to be able to understand the needs of the Vulnerable Child and to gain the ‘historical awareness’ regarding the development of his prominent schemas. For other cases, in which the Vulnerable Child plays a more manifest role, this might not be necessary. Apart from performing case studies, we recommend conducting interviews with patients regarding their experiences of automaticity. When we search literature on automaticity and drug use, this yields thousands of papers. However, most of this research makes inferences regarding automaticity based on brain imaging or computer-based tasks [e.g., (2, 59–61)]. It is highly uncommon for automaticity researchers to directly ask patients about their experiences of automaticity and the way these experiences change during therapy. Fredericks and Samuel (62) interviewed patients with substance abuse disorder about their drug use, recovery, relapse and ongoing struggles. They infer that using drugs, for these patients, had become automatic (in the sense that it was unintentional), but it is unclear how these experiences changed over time. It is somewhat surprising that so little research is done in which patients are directly asked about their experiences of automaticity, since evidence exists that people are able to self-report automatic behaviors (63–65). Similarly, interviews with clinicians can also provide valuable insights not only regarding changes in automaticity, but also regarding the ways in which they (intentionally or unintentionally) use information about automaticity to guide their therapeutic decisions.

Second, empirical evidence for the automatic activation of schemas and schema modes so far is lacking. However, a myriad of methods is available to examine awareness, control, efficiency and speed in quantitative research settings within the context of addiction. First, we suggest triggering a particular schema or schema mode by, for instance, presenting the participant with a relevant movie clip (see, for instance, (66)). Subsequently, we suggest it is possible to measure the automaticity of the triggered schema mode using a range of different tasks. For instance, subliminal priming techniques can be used to examine the effect of unconscious stimuli on behavior [e.g., (67)]. In these tasks, subconsciously presented neutral or salient primes are presented before a target. If the salience of a prime affects the responses to the target, it is assumed that the primes unconsciously affect behavior. Furthermore, approach/avoidance tasks [e.g., (68)], stop-signal tasks and go-no go tasks [e.g., (69)] can be used to examine elements of controllability. For instance, in a stop-signal task, participants are required to respond to specific stimuli, unless a stop-signal is presented. Research shows that it is more difficult to stop when particular salient stimuli are presented [e.g., (70)]. The efficiency of activation can be measured with attention tasks which compare the efficiency with which salient stimuli compared to neutral stimuli are processed [e.g., (71)] or by examining the effect of mental load on the processing or responding to particular stimuli (72). Finally, speed can be examined within a lexical decision task in which participants are presented with words and non-words. Results generally show that participants are slower to respond to salient words compared to neutral words (e.g., Gawronski & Bodenhausen).

When we simply replace the salient stimuli in these tasks with schema-related cues, they can be adapted to examine the automaticity of schema modes. This would allow us to assess different automaticity criteria during different times within the therapeutic process, giving us valuable information about changes in automaticity.

### Implications for automaticity in addiction

Our analysis of the role of automaticity in ST for substance use disorder is the first venture into this topic, and it is important to note that we cannot capture the complexity of automaticity in all its depth. First, we have not fully addressed the complex ways in which different automaticity criteria overlap and influence each other (17). Second, some theorists might suggest that there are different, more or fewer criteria of automaticity [e.g., (18, 73, 74)]. Third, Moors (17) makes a distinction between different levels of analysis: the observable level, the hidden level, and the brain level. Our analysis is mostly concerned with the observable level: we make inferences about schema modes and the extent to which they occur automatically by examining observable inputs (e.g., triggers) and outputs (e.g., behaviors that are in line with specific schema modes). Second, we concern ourselves with the hidden level: we make inferences about subprocesses that are not directly observable (e.g., the way in which different schema modes affect each other).

However, we have not discussed what happens at a brain level. It is important to note that a critique on automaticity theories or habit theories on addiction, is inherently also a critique on the brain disease model of addiction [BDMA; e.g., (75)] that poses that drug use causes long-term changes in specific brain regions, resulting in pathological (and automatic) drug-seeking behavior. Whereas automaticity theories draw direct links between automatic, stimulus-driven behaviors and the brain [e.g., (34, 35)] we do not make assumptions about how the development of psychopathology in general and specifically substance abuse is related to changes in specific brain regions. ST could thus not only provide an alternative automaticity model of addiction, because it regards automaticity in a more complex way (i.e., automaticity is not about habits, but about the automatic activation of schema modes), it also offers a more comprehensive and differentiated perspective on addiction than simplistic attributions of ‘brain disease’. Instead, ST helps seeing addiction as the result of childhood trauma. Whereas this trauma is likely to result in specific brain changes [e.g., (76)] we suggest that the therapeutic focus should not be on providing biomedical solutions but on trauma and healing. This idea is, to some extent, in line with a recent critique of researchers who similarly reject habit-theories of addiction, and is in favor of a model that focuses on socioeconomic deprivation and related issues as an explanation of drug abuse (4, 77). Whereas a thorough critique of the BDMA is outside the scope of this article, we hope that our ideas can fuel a discussion on this topic.

## Conclusion

In this article, we suggest that the understanding of the schema mode concept, especially when focused on severe addiction accompanied by personality disorders, might benefit from studying it from an automaticity perspective. Doing so might help comprehending the persistent nature of schema modes and the consecutive stages of change a schema mode goes through during successful ST. Further, introducing the schema mode concept to theories on automaticity in addiction, helps understanding why addictive behaviors are so hard to change and might provide tools for addiction treatment. Our exploration on the role of automaticity is the first of its kind and can be considered as an invitation to other researchers to continue this investigation to further develop a theoretical framework that is rooted in both ST as well as in automaticity, in the sense that it considers the effects of a history of trauma and the complexity of automaticity. Eventually, our line of thought might help the treatment of individuals suffering from addiction and co-morbid personality disorders.

## Data availability statement

The original contributions presented in the study are included in the article/supplementary material, further inquiries can be directed to the corresponding author.

## Author contributions

MB and HT wrote the manuscript. All authors contributed to the article and approved the submitted version.
